# Profiling Rest Intervals between Sets and Associated Factors in Resistance Training Participants

**DOI:** 10.3390/sports6040134

**Published:** 2018-10-30

**Authors:** Wellington Silva, Ricardo Viana, Douglas Santos, Rodrigo Vancini, Marília Andrade, Claudio de Lira

**Affiliations:** 1 Laboratório de Avaliação do Movimento Humano, Faculdade de Educação Física e Dança, Universidade Federal de Goiás, Goiânia 74690-900, Brazil; wellingtonedfisica2@gmail.com (W.S.); vianaricardoborges@hotmail.com (R.V.); datsantos@uneb.br (D.S.); 2 Colegiado de Educação Física da Universidade do Estado da Bahia, Campus X, Teixeira de Freitas, Bahia 45992-255, Brazil; 3 Centro de Educação Física e Desportos, Universidade Federal do Espírito Santo, Vitória 29075-910, Brazil; rodrigoluizvancini@gmail.com; 4 Departamento de Fisiologia, Universidade Federal de São Paulo, São Paulo 04023-901, Brazil; marilia1707@gmail.com

**Keywords:** intensity, exercise, recovery

## Abstract

The aims of this study were: (1) to report on and analyse rest interval (RI) control between sets adopted by resistance training (RT) participants; (2) to evaluate how RT participants control RIs; and (3) to describe the factors associated with controlling RIs, such as, gender, RT experience, and professional guidance. Four hundred and fifteen volunteers (198 women and 217 men) answered a questionnaire about RI control. Among the participants, 89.9% (n = 373) reported receiving guidance during physical training, of which 74.5% (n = 278) received instruction from a sports and exercise professional. The proportion of subjects that reported controlling RIs was 71.6% (n = 297). Most subjects that reported controlling RIs (95.0%, n = 282) reported adopting an RI of 60 s or less. There is no association of RI control with gender and professional guidance. The RI adopted by most of the participants might be considered short (<60 s), which is not in line with most RT guidelines. The results of the current study could be used to improve attitudes toward RT.

## 1. Introduction

Resistance training (RT) refers to a specialized method of physical conditioning that involves the progressive use of a wide range of resistive loads, different movement speeds, and a variety of training modalities, including weight machines, free weights (barbells and dumb-bells), elastic bands, medicine balls, and plyometrics [1]. Therefore, RT is a combination of static and dynamic contractions involving shortening [2] and lengthening of skeletal muscles (stretch-shortening contraction) or specialized movements that utilize the potentiation effect of the lengthening movement (for example, plyometrics, stretch-shortening cycle) [3].

The manipulation of programme variables such as exercise selection, training volume, training intensity, range of motion, movement velocity and rest interval (RI) between sets during RT are an integral part of a successful exercise prescription, and careful manipulation of these RT variables is vital for optimal results and preventing injuries [4,5,6]. In this context, the RI between sets has been considered an important variable due to its effects in terms of resistance exercise outcomes [4,5,6,7,8]. An adequate RI is necessary to offset the detrimental effects of fatigue and facilitate muscle recovery, allowing performance to be maintained or even improved in subsequent series [7,9].

Many studies have investigated the effects of RI on muscle variables, such as strength, power, and endurance in athletes, healthy subjects, and clinical populations from both sexes [10,11] as well as metabolic [12,13], neural [14], endocrine [13,15], cellular [16], and molecular [17] effects. Briefly, most studies and exercise guidelines suggest that at least 2 min should be adopted for increasing muscle strength and hypertrophy [7,18,19] and that shorter rest intervals (<60 s) might have a negative impact on muscle performance [10,20] in young and old adults.

In light of the importance of RIs, a rigid control of this variable is usually performed in research centres in order to follow the rigour of the scientific method. However, in gym or other exercise facilities, RT participants might not be aware of the importance of controlling RIs. Therefore, it is reasonable to suppose that there is a proportion of RT participants that do not control RIs, or control them inappropriately. Thus, the primary purpose of this study was to report on and analyse RI control adopted by RT participants. The secondary purposes of this study were to evaluate how RT participants control RIs and to describe factors associated with RI control, such as gender, RT experience, and professional guidance.

## 2. Materials and Methods

### 2.1. Study Design

A descriptive and cross-sectional study design was used for this study. To accomplish the purposes of the study, we developed a short questionnaire, described in detail below. The questionnaire included questions about practices adopted by RT participants.

### 2.2. Subjects

A total of 415 RT participants of both sexes (198 women and 217 men) were recruited for this study. The subjects were recruited through direct contacts in fitness centres located in the metropolitan region of Goiânia (situated in the Brazilian Midwest) and Vitória (situated in south-eastern Brazilian). Only individuals engaged in RT were included in the study. Subjects that were under 18 years of age and were personal trainers or coaches were excluded. 

All participants were informed of the intent, procedures, benefits and risks of the study and signed an informed consent form before data collection. All procedures involved in this study were approved by the University Ethics Committee (protocol number: 2.077.532) and followed the principles outlined in the Declaration of Helsinki. 

### 2.3. Survey

After an extensive literature review, a questionnaire was designed to evaluate attitudes toward RT variables. The questionnaire was composed of 19 simple, close-ended questions. The questions were from two domains: personal (8 questions) and RT (11 questions). The RT domain contained questions about professional guidance, the academic training of the exercise advisor, the main objective of RT practice, experience with RT, weekly frequency and volume, knowledge about RI influence on training outcomes, and the length of the RI adopted. All questionnaires were checked for correct completeness. Although the reliability and validity of the questionnaire were not determined, this survey was constructed in accordance with previous recommendations [21,22,23]. The questionnaire used is available as supplementary material.

### 2.4. Statistical Analysis

Data were analysed through the Statistical Package for the Social Sciences (SPSS) version 20.0 (IBM Corp., Armonk, NY, USA). Descriptive statistics were used to analyse the findings (mean, standard deviation, and absolute and relative frequencies). A chi-square test (χ^2^) was applied to evaluate the association of gender and main objective with RT and RT experience. Furthermore, χ^2^ was applied to evaluate the association between RI and the presence of professional orientation and experience with RT. Fisher’s exact test was applied to evaluate the association between RI and sex. The Mann–Whitney test was applied to compare age, body mass, height, body mass index (BMI), and training frequency between the sexes since these data were not normally distributed according to the Kolmogorov–Smirnov test. Statistical significance was set at *p* < 0.05.

## 3. Results

Table 1 shows the characteristics of subjects. There was no significant difference in age between sexes. 

Descriptive data about the RT protocol reported by subjects are shown in Table 2. On average, the participants performed 3.9 ± 1.3 sessions of RT per week, and there was no statistically significant difference between sexes (*p* = 0.085). The chi-square test revealed a significant association between sexes and the main objective of RT (*p* = 0.003). Out of the 415 participants, the most frequent reason to practice RT was to obtain muscle hypertrophy for men and to achieve weight loss and enhance health/quality of life for women. The chi-square test also revealed a significant association between sexes and RT experience (*p* = 0.002). Overall, those subjects who were more experienced with RT controlled more RIs (47.0% of men and 36.9% of women with RT experience >12 months). Most of the participants (61.2%) trained for at least 6 months.

Most participants (71.6%, n = 297) reported using a method to control RIs between sets. The Fisher’s exact test revealed no association between sexes for RI control (*p* = 0.12). Among those that admitted to controlling RIs, 24.3% (n = 72), 26.7% (n = 79), 13.3% (n = 40), 24.6% (n = 73), 2.7% (n = 8), and 1% (n = 3) admitted to adopting RIs of 15 s, 30 s, 45 s, 60 s, 90 s, and more than 90 s, respectively. Therefore, 88.9% of the participants that reported controlling RIs adopt an RI of up to 60 s. Only 28.4% (n = 118) reported not controlling RIs. In addition, among those that admitted to controlling RIs, 29.0% (n = 86) reported using a wristwatch with a stopwatch or timer, 23.2% (n = 69) reported using a rating perceived exertion (RPE) scale, 19.9% (n = 59) reported using a mobile phone with a clock, stopwatch or timer, 20.2% (n = 60) reported using a gym clock, 2.4% (n = 7) reported that coaches carried out control and 5.4% (n = 16) used other types of RI control. Specifically, in terms of who reported the use of an RPE, most of them (91.2%) reported using an RI of up to 60 s.

We also found that 89.9% (n = 373) of the participants received professional exercise advice. Fisher’s exact test revealed no association in controlling RIs between those who had and those who had not received professional guidance (*p* = 0.55). The majority (68.4%, n = 284) of the subjects knew that controlling RIs influences exercise intensity. Fisher’s exact test revealed a significant association between knowing that RI influences exercise intensity and a greater control of RIs (*p* < 0.001). The chi-square test revealed a significant association between RT experience and controlling RIs (*p* < 0.002). The highest proportion of people reporting RI control was in the group with ≥12 months of RT experience, followed by <3 months, ≥6 and <12 months, and ≥3 and <6 months. A total of 81.0% (n = 336) of the subjects reported that they were reaching their goals with RT. There was a significant association between reaching goals and control of RIs (*p* < 0.001). Finally, the chi-square test revealed a significant association between RI control and the main objective of resistance training (*p* = 0.049), while Fisher’s exact test did not reveal a significant association between the presence of non-communicable disease and RI control (*p* = 0.491) (Table 3). These results are shown in Table 3.

## 4. Discussion

The current study aimed to report and analyse RI control by RT participants. The majority of the participants reported controlling RIs. Among those that admitted to controlling RIs, the most frequently adopted RIs were between 15 and 60 s. Whilst scientific literature presents data suggesting that shorter RIs might be useful in healthy middle-aged women [24] and studies in trained men suggested that reduced RIs produce similar gains in muscle hypertrophy and performance [25], the current scientific literature oriented to muscle strength and hypertrophy suggests applying higher RIs [10]. We also found that there is a significant association between the main objective of RT and sex, RT experience and sex, RI control, and knowing that RI control influences exercise intensity, RT experience, and RI control, which is achieving objectives and RI control, and the main objective of resistance training and RI control. 

Current evidence suggests that an RI of at least 2 min must be utilized for increasing muscle strength and hypertrophy [7]. In a recent meta-analysis, Grgic et al. [6] reported that, although gains in muscular strength can be achieved with short RIs (<60 s), it seems that a longer duration (>120 s) and short to moderate RIs (60–120 s) are required to maximize strength gains in resistance-trained and untrained individuals. Senna et al. [18] compared repetition performance in 1-, 3-, and 5-min RIs of multi- and single-joint exercises. The exercises were performed for five sets with 10 repetition maximum (RM) loads by men with more than one year of RT experience. The authors reported consistent declines in performance (relative to the first set) for all rest conditions (i.e., 1, 3, and 5 min). The authors suggested that RI lengths of between 3 and 5 min must be adopted regardless of the type of exercise (i.e., multi- or single-joint exercises) in order to maintain the number of repetitions over sets. 

In the same vein, Bottaro et al. [26] found that non-resistance-trained young men may require an RI of a minimum of 120 s to recover full peak torque and total work in the knee extensor. Willardson and Burkett [10] compared 30-, 60-, and 120-s RIs in the number of repetitions completed by RT-experienced college-age men for the bench press and squat over five sets with a constant 15-RM load, and showed that both exercises performed with 120 s of RI resulted in a significantly greater number of repetitions than 30 s of RI. Theou, Gareth, and Brown [20] showed that short RIs (i.e., 15, 30, and 60 s) influenced the loss of muscle strength during RT sessions in women. The authors also showed significant declines in peak torque between the first and the third series with RIs of 15 to 30 s in the knee extension. In addition, Robinson et al. [27] showed that the use of short RIs may compromise the increases in muscle strength, and Figueiredo et al. [28] reported that the use of 60-s RIs between sets and exercises is associated with greater cardiac stress. Therefore, the RI adopted by subjects surveyed is not in line with the current scientific evidence. Surprisingly, the majority of subjects interviewed (89.9%) reported that they received instruction provided by a health professional with an academic degree in sports and exercise. It is reasonable to assume that these health professionals did not have the correct knowledge about RT exercise. Previously, some authors demonstrated that sports and exercise professionals and coaches have misconceptions about exercise prescription [29,30] and clinical situations [31,32,33]. This situation is alarming, because, at least theoretically, training supervision by specialized professionals should help to control important training variables such as load, RIs, and exercise technique, and to provide motivation and psychological reinforcement [34].

These findings are worrisome, since being supervised does not influence RI control nor guarantee that RIs adhere to the recommended guidelines, which reinforces the need for continued education for health professionals [29]. A total of 2.4% of the participants reported that coaches control their RIs. It is no coincidence that this portion of the sample was supervised by personal trainers, who usually have a particular educational level [35].

Therefore, given that the majority of the participants of the present study were engaged in RT in order to obtain muscle hypertrophy and enhance health/quality of life, and adopted RIs of ≤60 s, despite the fact that most of them reported achieving their goals (Table 3), it is reasonable to recommend an increase in RIs to avoid a substantial reduction in total work volume when the sets are performed until momentary muscle failure without adjusting the workload. In addition, if the RT participant uses a short RI, they will probably need to reduce the workload.

We also found a significant association between knowing that RI interfered with exercise intensity and its control. This was probably due to the subjective feeling that RT sessions become harder when the RI is lower. Furthermore, among those subjects that reported that they did not control RIs, 37.3% reported that they are not meeting the objectives of RT, while only 11.8% of participants that reported controlling RIs are achieving the objectives. These results show clearly that controlling RIs, to a greater extent than other variables related to RT, is a key factor for the success of RT.

In the context of RT purposes, it is very common that subjects report enhanced hypertrophy or health/quality of life as the main purpose of RT [36,37]. In our study, 24.8% of the subjects surveyed reported enhanced hypertrophy as being their main aim and 27.0% reported health/quality of life. Specifically, we found a significant association between the main objective of RT and sex. Briefly, 31.3% of male subjects surveyed reported muscle hypertrophy as the main objective of RT, while 17.7% of female participants reported that they performed RT to improve their health/quality of life and 21.7% of female participants reported that they performed RT for weight purposes. Furthermore, we found that subjects that reported improved health/quality of life as their main purpose control RIs more than those that reported hypertrophy, perhaps because the former performed exercises in order to manage a non-communicable disease. 

Moreover, a significant association was found between RT experience and control of RIs. In other words, more trained/experienced participants were more likely to control RIs, and it is known that increasing strength and muscle mass over the RT sessions becomes increasingly difficult [38]. Thus, controlling all RT variables (e.g., RI between sets and exercises) becomes increasingly important. Therefore, it is reasonable to suppose that RT participants assume a positive attitude toward RT over time, especially those that are highly engaged.

Another interesting finding of the current study is that among those that admitted to controlling RIs, 29.0% reported using a wristwatch (a device that can be constituted by a stopwatch and/or a timer), which is, in practice, the easiest way to control RIs. The use of a wristwatch is a common method of time-keeping in modern-day life and does not present a risk to the user. Although a smartphone has a stopwatch and/or timer, we believe that a smartphone offers several apps with the potential to distract the user during the training session, such as social media (e.g., Facebook, Instagram, WhatsApp, and YouTube), which are very popular among young adults [39]. Indeed, Villanti et al. [39] found that mobile devices are a primary channel for social media. Although, to the best of our knowledge, there was no research that evaluated the use of smartphones in gym/exercise facilities, it is reasonable to assume that RT participants use this device during training sessions. This hypothesis gains strength if we consider that thousands of health applications (including fitness apps) are available for free or at a very low cost [40]. With regard to the gym clock, we believe that this strategy is limited, since a gym clock rarely corresponds to a stopwatch, and generally, there are a limited number of throughout the gym.

RPE involves the ability to detect and interpret organic sensations while performing exercises [10]. This method has been used to measure the level of effort that is felt during exercise at a given intensity and is a versatile, simple, and well-accepted marker of exercise training load [41]. RPE has more traditionally been used to evaluate exertion and intensity in an aerobic exercise. However, investigations have emerged supporting its utility to measure both set and session intensity for RT in different populations [4]. Theoretically, RPE can be used as a tool to monitor and adjust the training intensity in a way that can elicit an appropriate training stressor. However, its use as a marker of recovery has not been validated. In our study, 23.2% (n = 69) reported using the RPE scale and 91.2% of subjects surveyed reported that RPE control is equivalent to an RI of up to 60 s. As previously discussed in this manuscript, this RI is not adequate given the current scientific evidence.

Finally, future observational research must be conducted to verify the real RI adopted by RT participants and how they are actually controlling the RI. To this end, we suggest that RT participants be observed during training sessions.

## 5. Study Limitations

As with all studies employing questionnaires, the present results rely on the honesty and level of recall of the respondents. The reliability and validity of the instrument used to gather the data for this study have not been determined. In addition, the answers to some questions were of a ‘yes’ or ‘no’ type; therefore, we were not able to assess an intermediary answer. Despite these limitations, the results are still meaningful and do not prevent conclusions being drawn from the study.

## 6. Conclusions

In conclusion, the results of the present study show that there are associations between the control of RIs with RT experience and personal goals. However, there is no association between gender and professional advising. In addition, 95.0% of the surveyed subjects admitted to adopting between 15 and 60 s of RI and, although information about training intensity is not available, the use of short RI might interfere with training performance. Therefore, it is desirable that sports and exercise professionals/coaches be cognisant of the dynamic and continually evolving nature of scientific evidence and, therefore, they should seek continuing education programmes to maximize the quality of the service provided by them, especially in regard to RT. Although the majority of participants surveyed were counselled by these professionals, we found that the RI adopted was not adequate.

## Figures and Tables

**Table 1:** Characteristics of all participants (mean ± standard deviation).

Characteristic	Men (n = 217)	Women (n = 198)	*p*	Total (n = 415)
Age (years)	31.3 ± 11.9	34.4 ± 11.2	0.129	32.1 ± 11.8
Height (m)	1.77 ± 0.07	1.63 ± 0.06	<0.001	1.70 ± 0.1
Body mass (kg)	80.2 ± 13.3	64.1 ± 10.0	<0.001	72.6 ± 14.3
BMI (kg·m^−2^)	25.6 ± 3.6	24.0 ± 3.4	<0.001	24.9 ± 3.7

**Table 2:** Characteristics of resistance training reported by subjects.

Characteristics	Men	Women	Total	*p* Value
	(n = 217)	(n = 198)	(n = 415)	
Sessions per week ^1^	4.0 ± 1.2	3.9 ± 1.2	3.9 ± 1.3	0.085 ^3^
Main objective of resistance training ^2^				
Muscle hypertrophy	31.3% (68)	17.7% (35)	24.8% (103)	
Muscle definition	12.0% (26)	15.2% (30)	13.5% (56)	
Health/quality of life	24.0% (52)	30.3% (60)	27.0% (112)	0.003 ^4^
Recreation/socialization	7.8% (17)	5.6% (11)	3.1% (13)	
Physical conditioning	6.0% (13)	6.1% (12)	6.0% (25)	
Muscle strengthening	7.4% (16)	3.5% (7)	5.5% (23)	
Weight loss	11.5% (25)	21.7% (43)	16.4% (68)	
Resistance training experience ^2^				
0–3 months	13.8% (30)	24.2% (48)	18.8% (78)	
3–6 months	21.2% (46)	18.7% (37)	20.0% (83)	0.002 ^4^
6–12 months	18.0% (39)	20.2% (40)	19.0% (79)	
≥12 months	47.0% (102)	36.9% (73)	42.2% (175)	

^1^ Data are shown as mean ± standard deviation. ^2^ Data are shown as relative frequency (absolute frequency). ^3^ *p* value from Mann-Whitney test. ^4^ *p* value from chi-square test.

**Table 3:** Control of rest interval between sets according to gender, resistance training experience, exercise advising, and meeting the goals.

Variable	Control of Rest Interval between Sets		*p*
	Yes (n = 297)	No (n = 118)	
Gender			0.12 ^#^
Men	54.2% (161)	47.5% (56)	
Women	45.8% (136)	52.9% (62)	
Do you know that the rest interval between sets influences the exercise intensity?			
Yes	74.1% (220)	54.2% (64)	<0.001 ^#^
No	25.9% (77)	45.8% (54)	
Resistance training experience			
<3 months	21.9% (65)	11.0% (13)	0.002 *
≥3 and <6 months	16.2% (48)	29.7% (35)	
≥6 and <12 months	17.8% (53)	22.0% (26)	
≥12 months	44.1% (131)	37.3% (44)	
Do you receive professional guidance?			0.55 ^#^
Yes	89.9% (267)	89.8% (106)	
No	10.1% (30)	10.2% (12)	
Are you reaching your goals?			
Yes	88.2% (262)	62.7% (74)	<0.001 ^#^
No	11.8% (35)	37.3% (44)	
Main objective of resistance training			
Hypertrophy	22.6% (67)	30.5% (36)	0.049 *
Muscle definition	12.5% (37)	16.1% (19)	
Health/quality of life	30.0% (89)	19.5% (23)	
Recreation/socialization	8.4% (25)	2.5% (3)	
Physical conditioning	5.1% (15)	8.5% (10)	
Strength	5.7% (17)	5.1% (6)	
Weight loss	15.8% (47)	17.8% (21)	
Presence of non-communicable disease			
Yes	17.2% (51)	17.8% (21)	0.491 ^#^
No	82.8% (246)	82.2% (97)	

Data are shown as relative frequency (absolute frequency). * Chi-square test. ^#^ Fisher’s exact test.

## Supplementary Materials

The following are available online at http://www.mdpi.com/2075-4663/6/4/134/s1.
