# Supplementary material for: Profiling Rest Intervals between Sets and Associated Factors in Resistance Training Participants
Source: Sports (Basel). 2018 Oct 30;6(4):134. doi: 10.3390/sports6040134 (PMC6316470; doi:10.3390/sports6040134)
Supplement: Supplementary file 1 [file sports-06-00134-s001.pdf]

UFG

Federal University of Goiás
Faculty of Physical Education and Dance
Human Movement Assessment Laboratory

Questionnaire

Gym/training place: ____Date_________ ID: _______

A) Personal data

1) Name: ____
2) Sex: ☐ Male ☐ Female
3) Profession: _______________________________________
4) Body mass: ____kg
5) Height: ______m
6) Date of birth: ____/_____/_______
7) Education level:
   ☐ Incomplete elementary School ☐ Complete elementary school ☐ Incomplete high school
   ☐ Complete high school ☐ Incomplete higher education ☐ Complete higher education
   ☐ Incomplete postgraduation ☐ Complete postgraduation
   ☐ Specialization
   ☐ Master degree
   ☐ Doctorate degree

8) Do you have any of the following diseases?
   ☐ Diabetes mellitus ☐ Arterial hypertension ☐ Hypercholesterolaemia
   ☐ Depression ☐ Epilepsy ☐ Obesity
   ☐ Cardiopathy ☐ Other: Please specify ______________ ☐ I have no disease

B) Physical activity

9) In what period of the day do you train? (If necessary, select more than one alternative)
   ☐ Morning ☐ Afternoon ☐ Night ☐ Dawn
10) Do you receive professional advice? ☐ Yes ☐ No
11) Do you receive advice from a *personal trainer*? ☐ Yes ☐ No
12) What academic training has your advisor had?
   ☐ Physical education student ☐ Physical education professional
   ☐ Doctor ☐ Physiotherapist.
   ☐ I don't know ☐ Other. Please specify________________________

13) In order of importance (1 to 8), what are your goals with resistance training (1 being the most important goal and 8 being the least important goal)?
   ( ) Muscular hypertrophy ( ) Muscle definition ( ) Health/quality of life
   ( ) Recreation/socialization ( ) Weight loss ( ) Physical conditioning
   ( ) Muscle strengthening

14) Do you think you are achieving your main objective with resistance training?
   □ Yes □ No. If no, whyvnot? ____.

15) Do you do any other type of physical activity besides resistance training?
   □ No □ Yes. If yes, please specify____.

16) How long have you been practising resistance training (consider the current period)?
   □ 0–3 months □ 3–6 months □ 6–12 months □ ≥12 months.

17) How many days a week do you practise resistance training?
   ☐ 1 day ☐ 2 days ☐ 3 days ☐ 4 days ☐ 5 days ☐ 6 days ☐ 7 days

18) How long is each resistance training session?
   ☐ ≤ 30 minutes ☐ 30 to 60 minutes ☐ 60 to 90 minutes
   ☐ > 90 minutes ☐ I do not check the duration of my resistance training session.

19) Generally, how many exercises do you do in your resistance training sessions?
   ☐ 1 to 5 exercises ☐ 6 to 10 exercises ☐ 11 to 15 exercises
   ☐ 16 to 20 exercises ☐ ≥ 20 exercises

20) On average, how many sets do you perform in each resistance training exercise?
   ☐ 1 set ☐ 2 sets ☐ 3 sets ☐ 4 sets ☐ ≥ 5 sets

21) Do you usually control the rest interval between sets?
 No Yes

22) On average, what is the rest interval between sets?
 15 seconds 30 seconds 45 seconds
 60 seconds 90 seconds ≥ 90 seconds
 I do not control the rest interval between sets

23) Did you know that the rest interval between sets influences the intensity of the resistance training exercise?
 No Yes

24) Did you know that the rest interval between sets may influence the results obtained through resistance training practice?
 No Yes

25) Did you know that the rest interval between sets may differ according to the goals of the resistance training programme (muscle hypertrophy, muscle strength, weight loss...)?
 No Yes

26) How do you control the rest interval between sets?
 Wristwatch with stopwatch or timer Mobile phone with clock, stopwatch or timer
 Rating of perceived exertion The coach controls the rest interval between sets
 Gym clock I do not control the rest interval between sets
Other. Please specify____

27) Do you use a wristwatch during your resistance training session?
 No Yes

28) If not, why not?
 I do not have a wristwatch. I have a wristwatch, but I am not in the habit of using it
 It disturbs the resistance training performance Other. Please specify_______________________

29) If yes, why do you use your wristwatch during your resistance training session (if necessary, select more than one alternative)?
 To control the rest interval between sets Only as a garment accessory
 To avoid being late for another commitment (e.g., work or study) Other reasons. Please specify________________________

30) How do you rate the intensity of your resistance training session?
 Very, very light Very light Quite light
 Quite heavy Heavy Very heavy
 Very, very heavy
